# Bile Chemistry During Ex Situ Normothermic Liver Perfusion Does Not Always Predict Cholangiopathy

**DOI:** 10.1097/TP.0000000000004944

**Published:** 2024-02-27

**Authors:** Christopher J.E. Watson, Rohit Gaurav, Lisa Swift, Corrina Fear, Michael E.D. Allison, Sara S. Upponi, Rebecca Brais, Andrew J. Butler

**Affiliations:** 1 Department of Surgery, University of Cambridge, Addenbrooke’s Hospital, Cambridge, United Kingdom.; 2 The National Institute of Health Research Cambridge Biomedical Research Centre, Cambridge, United Kingdom.; 3 The National Institute for Health Research Blood and Transplant Research Unit at the University of Cambridge in collaboration with Newcastle University and in partnership with National Health Service (NHS) Blood and Transplant, Cambridge, United Kingdom.; 4 The Roy Calne Transplant Unit, Cambridge University Hospitals NHS Foundation Trust, Cambridge, United Kingdom.; 5 Department of Medicine, Cambridge University Hospitals NHS Foundation Trust, Cambridge, United Kingdom.; 6 Department of Radiology, Cambridge University Hospitals NHS Foundation Trust, Cambridge, United Kingdom.; 7 Department of Pathology, Cambridge University Hospitals NHS Foundation Trust, Cambridge, United Kingdom.

## Abstract

**Background.:**

Bile chemistry during normothermic ex situ liver perfusion (NESLiP) has been suggested to be an indicator of cholangiopathy. The normal range of biochemical variables in bile of livers undergoing NESLiP has not been defined, nor have published biliary viability criteria been assessed against instances of posttransplant nonanastomotic bile strictures (NASs).

**Methods.:**

The bile and perfusate chemistry of 200 livers undergoing NESLiP between February 1, 2018, and October 30, 2023, was compared. In addition, 11 livers that underwent NESLiP and later developed NAS were selected and their bile chemistry was also examined.

**Results.:**

In livers that did not develop cholangiopathy, concentrations of sodium, potassium, and chloride were slightly higher in bile than in perfusate, whereas the concentration of calcium was slightly lower. Bile was alkali and had a lower glucose concentration than perfusate. Cholangiocyte glucose reabsorption was shown to saturate at high perfusate concentrations and was more impaired in livers donated after circulatory death than in livers donated after brain death. Published criteria failed to identify all livers that went on to develop NASs.

**Conclusions.:**

A significant false-negative rate exists with current biliary viability criteria, probably reflecting the patchy and incomplete nature of the development of NASs in the biliary tree. The data presented here provide a benchmark for future assessment of bile duct chemistry during NESLiP.

## INTRODUCTION

Bile production by the liver is an indicator of liver health. With the introduction of normothermic ex situ liver perfusion (NESLiP), the quantity of bile produced was one of the first biomarkers to be suggested as an indicator of liver viability,^1^ and measurement of its rate of production was a feature of the first automated perfusion machines. Research has shown that bile ducts are not simple conduits for bile but are lined with cholangiocytes with absorptive and secretory properties that actively modify bile as it passes from hepatocyte to hepatic duct.^2,3^ Up to 40% of the bile in the hepatic duct is reported to be a consequence of secretory processes by cholangiocytes, something that can increase postprandial under the influence of hormones, such as secretin and gastrin.^2^ Research with animals and with human cholangiocyte organoids has suggested that there is heterogeneity of cholangiocyte function, as well as histological appearance, from canaliculi to hepatic duct.^2,4^ Thus, analysis of the composition of bile in the hepatic duct may reflect the function of multiple levels of cholangiocytes along the biliary tree and has been an area of interest for clinicians undertaking NESLiP who want to be able to predict which livers will and will not develop nonthrombotic ischemic cholangiopathy posttransplant.^5^

Among the secretory and absorptive processes undertaken by cholangiocytes are the uptake of glucose and secretion of bicarbonate and chloride, all of which can be evaluated in real time during NESLiP using a point-of-care blood gas analyzer. Several authors, including ourselves, have suggested biochemical thresholds indicating bile duct integrity and, therefore, minimal risk of posttransplant cholangiopathy but without extensive follow-up or concordance between reports, even between reports by the same authors.^6-11^

In this article, we describe the relationships between the electrolyte composition of bile and perfusate in 200 perfused livers, show the dynamic changes in bile composition as perfusion proceeds, and describe common patterns and variants. We also examine the bile chemistry of cases where cholangiopathy has occurred to evaluate published thresholds suggested to predict cholangiopathy.

## MATERIALS AND METHODS

As part of a clinical program we performed normothermic ex situ perfusion on donor livers that fell into the following groups, with a view to subsequent transplantation:

1. Livers in which there was a concern regarding viability, including all livers donated after circulatory death (DCD) that had not previously undergone normothermic regional perfusion (NRP).^12^
2. Recipients with adverse factors such as physiological instability in an urgent recipient or in whom surgery was expected to be difficult, and an extended preservation time would be advantageous.
3. Where logistics prevented the surgery from beginning in a timely manner.

### Liver Perfusion

All livers underwent normothermic machine perfusion using the metra (OrganOx, Oxford, United Kingdom). The perfusate comprised third-party donor red cells suspended in either Gelofusine (BBraun Medical Ltd) or 5% human albumin solution, together with supplementary magnesium, calcium, and amino acids, with additional sodium bicarbonate or tris-hydroxymethyl aminomethane (used if the sodium concentration was >142 mmol/L) to maintain a pH >7.2, and glucose when the perfusate glucose level fell <10 mmol/L (180 mg/dL). Before being placed on the metra, livers were flushed with Hartmann’s compound sodium lactate solution (Baxter, United Kingdom) to preload the liver and circuit with lactate and wash out the University of Wisconsin solution. When permitted, donor blood was washed on a cell saver (Haemonetics Ltd, Coventry, United Kingdom) to remove potassium and red cell debris before being added to the perfusate. The different chemistry of third-party donor-packed red cells of varying ages, together with the varying composition of effluent from the donor livers, resulted in different perfusate compositions despite our standardized protocol. Heparin, epoprostenol, taurocholic acid, and insulin were infused for the duration of the perfusion.

### Bile Duct Management During Transplantation

Biliary anastomosis was by direct duct-to-duct anastomosis without a T-tube unless the liver was a retransplant, the indication was primary sclerosing cholangitis, or there was a significant size discrepancy, in which case hepaticojejunostomy was performed, also without a stent.

### Bile Collection and Perfusate Chemical Analysis

The common bile duct was cannulated with a 12-French biliary catheter (T-Kehr type, Redax, Italy) during NESLiP. This drainage tube was divided outside the liver and connected to a 50 mL enteral drainage bag (GBUK Group, Selby, United Kingdom), excluding air, connected to a 3-way tap to facilitate frequent sampling. There was a dead space of 1.5 mL in the cannula between the bile duct and the collection bag. Bile and perfusate samples were collected at 45, 60, 90, 120, 150, and 180 min and hourly thereafter.

Bile and perfusate biochemistry was assayed on 1 of 2 point-of-care blood gas analyzers (Cobas b221, Roche Diagnostics, Indianapolis, IN, and Siemens RAPIDpoint 500e, Camberley, United Kingdom). Directly measured parameters included pH, pCO_2_, pO_2_, hemoglobin oxygen saturation, together with concentrations of sodium, potassium, chloride, calcium, glucose, and lactate. Bicarbonate and hydrogen ions are derived parameters in the analyzers; pH was measured in a range between 6 and 8 on the Roche machine and between 6 and 7.8 on the Siemens machine; values >8 on the Roche machine, or >7.8 on the Siemens machine were read as “high out of range,” but we have recorded them as 8 and 7.8, respectively.

### Livers With Cholangiopathy

All livers that developed nonthrombotic nonanastomotic strictures detected by magnetic resonance cholangiopancreatography undertaken in response to clinical symptoms or abnormal biochemistry between February 2018 and October 2023 were identified, and their biochemical parameters were reviewed. Livers developing strictures at the anastomosis or the first- and second-order ducts alone were not included.

### Statistics and Ethical Approval

Data were analyzed using PRISM version 10.0 (GraphPad Software Inc, San Diego, CA). Data were presented graphically as median and interquartile range. Comparison between groups was performed by the Kruskal-Wallis test for independent continuous variables.

The study was approved by the Human Biology Research Ethics Committee of the University of Cambridge (HBREC.2020.23).

## RESULTS

### Indications for Perfusion

Between February 1, 2018, and October 30, 2023, 304 livers underwent normothermic perfusion and 222 were transplanted. In most cases, perfusion started at the recipient center and at the donor hospital on only 4 occasions. Of the transplanted livers, 96 were from donation after brain death donors and 126 from controlled (Maastricht 3) DCD donors. Fifty-eight livers had previously undergone NRP and were perfused either because they failed NRP criteria or for logistical or recipient reasons. From these 222 transplants, 200 perfused livers were selected to determine a range of “normal” values, excluding cases of primary nonfunction (n = 2), those developing NAS, and those undergoing short perfusions. We acknowledge that perfusion of livers after a period of ischemia may not be completely representative of the in vivo state.

### Patient Outcomes and Cholangiopathy

The median follow-up was 620 d. One-year patient survival was 96%, 1 y death-censored graft survival was 94.6%, and 1 y transplant survival (graft survival not censored for death) was 92.4%. Twenty-nine patients developed nonanastomotic biliary strictures (NAS), 6 in association with hepatic artery thrombosis. Of the other 23, 11 developed hilar strictures affecting first- and second-order ducts alone, 1 developed a segment IV duct stricture in association with a split liver, and 11 developed widespread nonanastomotic strictures. Data on these 11 livers were presented individually (see also **Table S1, SDC,** http://links.lww.com/TP/C973) and compared with the 200 comparators in **Table S2** (**SDC**, http://links.lww.com/TP/C973).

### Perfusate Chemistry During Ex Situ Perfusion

**FIGURE 1.**
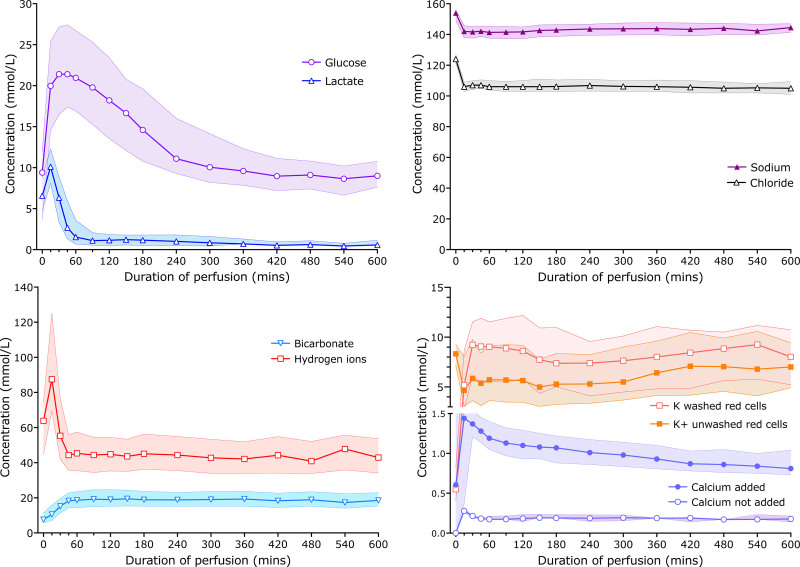
Median and interquartile ranges of perfusate analytes during normothermic perfusion of transplanted livers. Lactate falls rapidly and reaches baseline by 90 min. Glucose rises initially, plateaus, and then falls by zero-order kinetics until it reaches around 10 mmol/L (180 mg/dL), at which point an infusion of glucose begins and the concentration plateaus. Hydrogen ions fall initially and then plateau, and bicarbonate behaves reciprocally, rising before plateauing. Sodium and chloride both fall from the liver-free perfusate levels once the liver is connected to the circuit and remain constant throughout perfusion. Potassium concentrations are initially higher when perfusate contains unwashed red cells, compared with washed red cells; levels then fall slightly during NESLiP before plateauing. Where calcium is not added, the levels remain low and constant throughout perfusion. Where calcium is supplemented, the levels fall slowly throughout the perfusion period. NESLiP, normothermic ex situ liver perfusion.

Livers undergoing ex situ perfusion exhibited an initial high perfusate lactate, which usually fell to <1.5 mmol/L within 90 min, and glucose level that was initially high as a consequence of glycogen breakdown rose as lactate was converted to glucose and before glycogenolysis ceased, before falling by zero-order kinetics to around 10 mmol/L (180 mg/dL), at which point a glucose infusion was begun (Figure 1). Following reperfusion, the initial acidosis was countered by administration of sodium bicarbonate, so by 45 min, the hydrogen ion concentration was usually steady, as was the bicarbonate concentration. Sodium and chloride concentrations were also steady from a similar time point. At the start of the series, calcium was not added to perfusate; later, calcium was added at either the 15 or 30 min time points, after which it fell slowly through the course of perfusion; most recently, it has been added to the prime. There were also 2 different potassium profiles, depending on whether the red cells had been washed and excess potassium and cellular debris removed before they were added to the perfusate.

### Bile Chemistry

**FIGURE 2.**
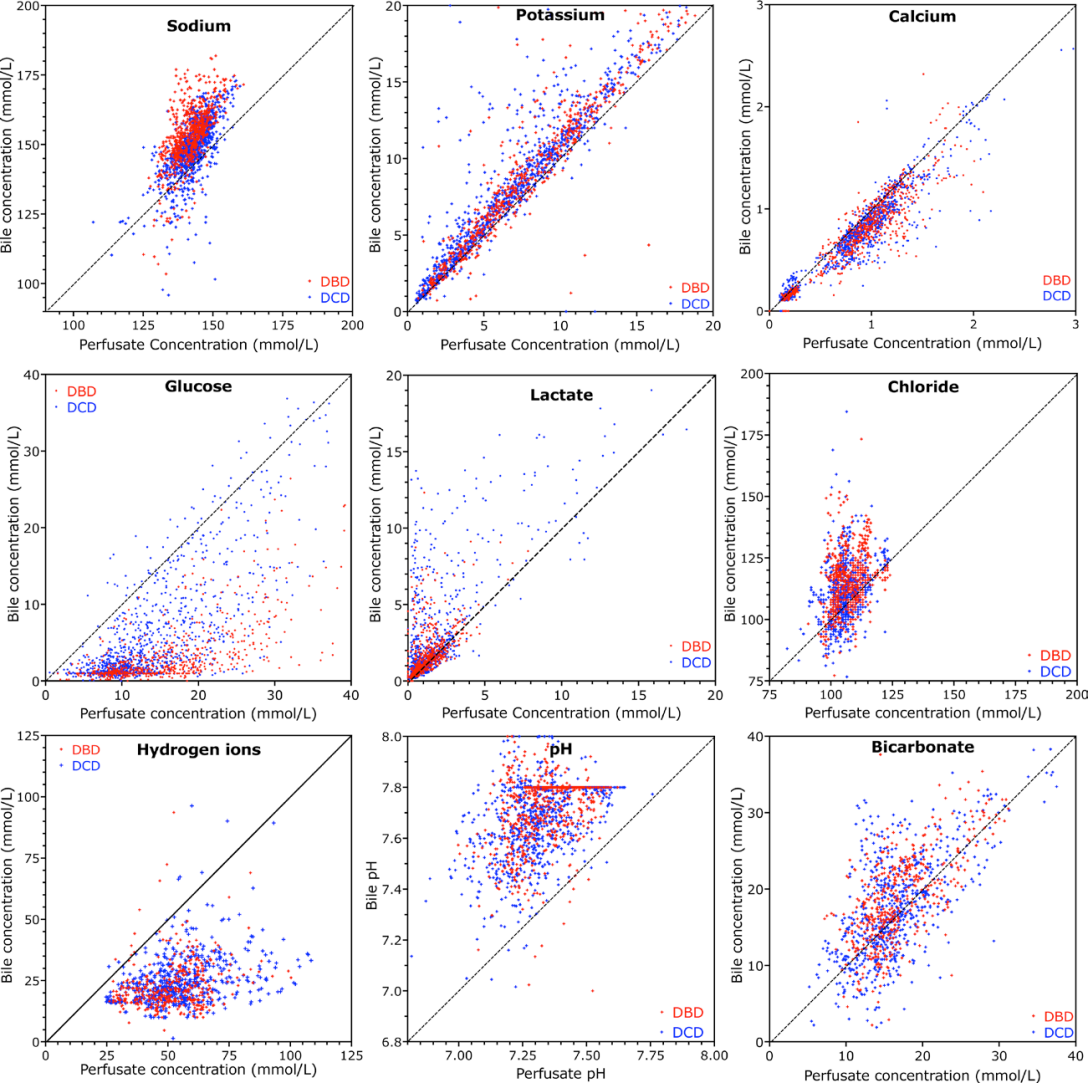
Scatter plots of bile concentrations at different perfusate analyte concentrations at all time points for 200 transplanted livers not developing cholangiopathy. Each dot represents a single bile concentration and corresponding perfusate concentration. DBD livers are colored red, and DCD are colored blue. Potassium is generally higher in bile than perfusate, as is sodium, although there is less difference in sodium concentrations. Calcium concentration in bile is generally slightly lower than in perfusate, whereas glucose is, for the most part, much lower. It is notable that only DCD livers have a bile glucose greater than perfusate glucose. The dead space in the bile duct and collecting cannula and relatively low bile flow rates account for why the bile glucose is recorded higher than perfusate, which has fallen in the period of time taken for bile to progress to the collection bag. Lactate is usually higher in bile, reflecting either the dead space in the bile cannula or anaerobic metabolism of the cholangiocytes. Chloride concentrations are generally higher in bile. DBD, donation after brain death; DCD, donation after circulatory death.

Figure 2 is a scatter plot displaying the concentrations of simultaneously measured analyte concentrations in bile and perfusate at the various time points during perfusions of all livers. In interpreting these, it should be remembered that there is a dead space of around 1.5 mL in the bile tube, and the rate of bile production was quite variable. Hence, the concentration of analytes in bile will represent changes before those measured in the perfusate at the same time; this will be more relevant if there is not much bile being produced. Differences in bile and perfusate concentrations caused by the time taken for bile to progress from the hepatocyte down the biliary tree and along the dead space in the drainage tube are particularly important when considering biliary glucose and lactate, which exhibit the biggest changes in concentration in the perfusate (Figure 1); it is less of an issue where the perfusate analytes were in steady state throughout perfusion, such as sodium and chloride for example.

**FIGURE 3.**
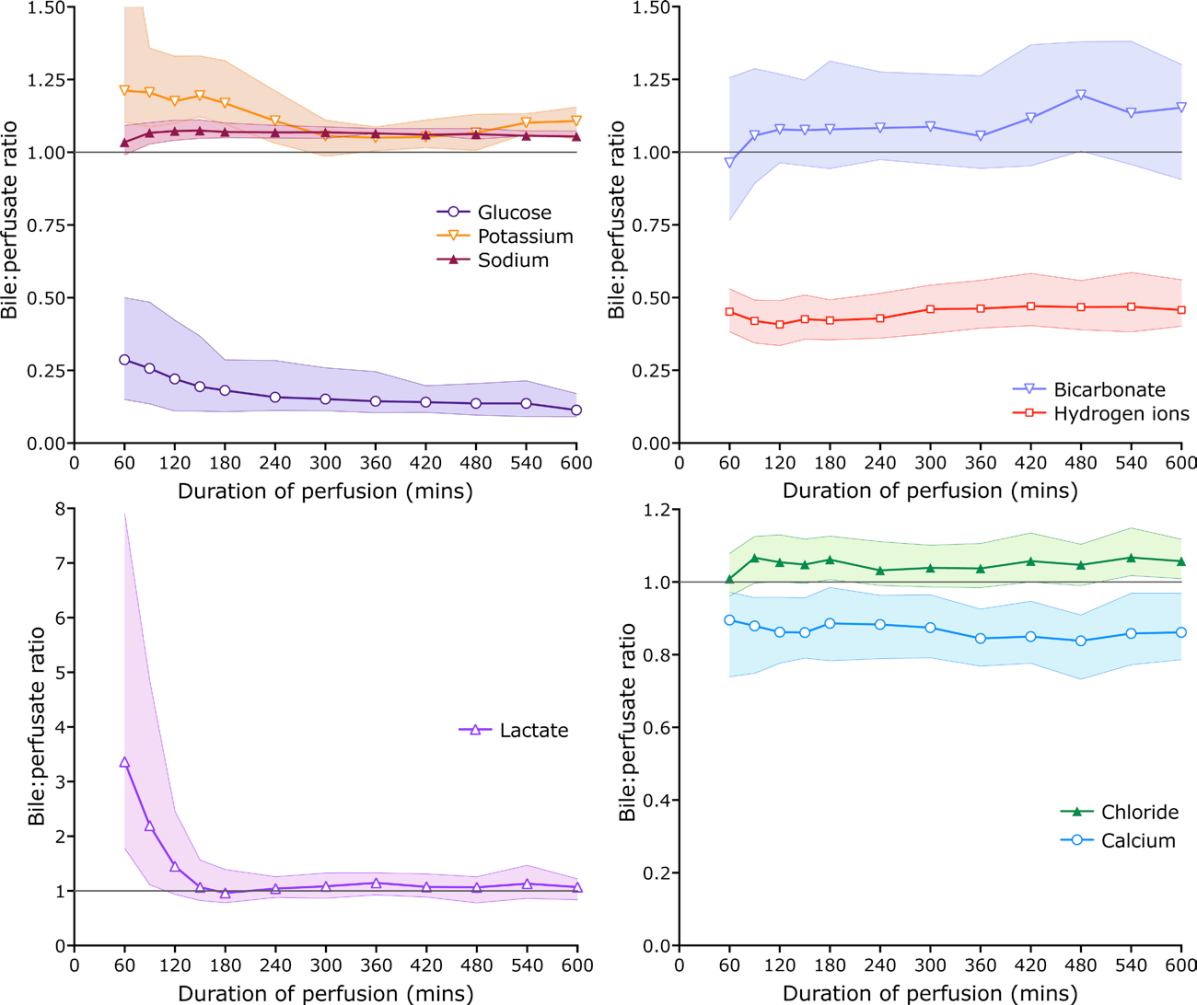
Bile:perfusate ratios of analytes of all transplanted livers during ex situ perfusion. Each graph shows the ratio of bile to perfusate concentrations and enables a ready appreciation of how bile chemistry differs from perfusate.

In general, sodium, potassium, chloride, and lactate are more concentrated in bile than perfusate, whereas the opposite is true of glucose and hydrogen ions (Figure 2). This is most clearly seen in Figure 3, which shows the median (±interquartile range) bile:perfusate concentration ratios, with the interquartile ranges shaded, of all the measured analytes in livers that were transplanted and which did not develop cholangiopathy.

### Sodium and Potassium

Fourteen of the 304 (4.6%) perfused livers had an initial bile sodium concentration under 110 mmol/L, which rose at later time points. Only 17 (5.6%) livers had a bile sodium concentration lower than perfusate at the end of perfusion, of which 8 had failed our criteria for transplantation for other reasons; in most cases the sodium concentration in bile was around 6% higher than perfusate (Table 1). The bile:perfusate sodium concentration ratio saw the least variation over time of all the analytes.

**TABLE 1.** Bile chemistry during normothermic liver perfusion

Analyte	Bile concentration relative to perfusate	Ratio relative to perfusates, median (IQR)	Notes
Sodium	Proportional to, but higher than, perfusate concentration	1.065 (1.043–1.089)	Enters bile via the sodium-bicarbonate cotransporter.
Potassium	Proportional to, but higher than, perfusate concentration	1.108 (1.037–1.208)	Tends to be higher early in perfusion. SK2 is a calcium-activated channel, which pumps potassium and provides an electrochemical force for pumping in chloride.
Calcium	Proportional to, but lower than, perfusate concentration	0.862 (0.774–0.952)	Mechanism of extraction of calcium from bile is unclear.
Glucose	Actively absorbed from bile	Dynamic depends on the capacity for resorption	Normally <1 mmol/L Resorption via the sodium-dependent glucose cotransporter. Initial bile concentrations are usually high as this pump’s capacity to recover glucose in bile is exceeded.
Lactate	Normally <1 mmol/L	Dynamic	Reflects both perfusate lactate and lactate produced locally by cholangiocytes. Not actively resorbed.
Chloride	Proportional to but higher than perfusate concentration	1.050 (1.000–1.125)	Actively pumped into bile by the cystic fibrosis transmembrane conductance regulator and then exchanged for bicarbonate by the AE2. In addition, there is probably a calcium-regulated chloride channel facilitating chloride entry into bile.
Bicarbonate	Actively pumped into bile	1.088 (0.951–1.283)	Machine results are calculated values based on pH and pCO_2_, which may, in part, account for a wide interquartile range. Bicarbonate is pumped into bile by the AE2 and also a sodium bicarbonate cotransporter.
pH	Normally >7.8	1.048 (1.037–1.060)	Reflects excess of bicarbonate in bile.
H^+^	Lower than perfusate	0.444 (0.367–0.537)	Machine results are calculated value based on pH. Reflects the addition of bicarbonate to bile.

See also Figures 2 and 3.

Summary data based on bile and perfusate chemistry of 200 livers that were transplanted and did not develop cholangiopathy. Individual measurements of bile and perfusate were taken simultaneously at 90 min and later during perfusions. Absolute values cannot be quoted because, for most analytes, the concentration depends on the perfusate concentration.

AE2, anion exchange pump 2; IQR, interquartile range.

There was a wide range of perfusate potassium concentrations, reflecting in part potassium released from dead hepatocytes, in part from retained preservation solution, and in part from third-party donor red cells used for perfusion. The concentration of potassium in bile was higher than perfusate for nearly all livers at nearly all time points. Beyond 5 h the ratio of bile:perfuate potassium was similar to that of sodium. The reason for the initial high bile:perfusate potassium ratio is not clear; retained preservation solution in the biliary tree is 1 possible explanation. There was no obvious biliary pathological correlation of abnormal sodium or potassium ratios.

### Lactate

Lactate rose in perfusate in the first 15 min, before being cleared from the circulation by 90 min in most livers. Lactate was initially much higher in bile, falling over the first 3 h to become similar to perfusate.

### Bile Acid/Base Balance

Bile is alkali, and during perfusions the concentration of hydrogen ions in the bile of transplanted livers that did not experience biliary complications was less than half that of perfusate (ie, the pH was higher). The hydrogen ion concentration fell after the initial bile sample for most livers. Bicarbonate was initially lower in bile than perfusate before rising until the median concentration ratio was higher than perfusate.

### Glucose

The median glucose concentration in bile was less than half that of the perfusate, falling further to a ratio <0.25 by 2 h. Figure 1 shows that bile:perfusate glucose concentration ratios are generally lower for livers from brain dead donors compared with those from DCD donors, although this was not significant with the numbers studied.

**FIGURE 4.**
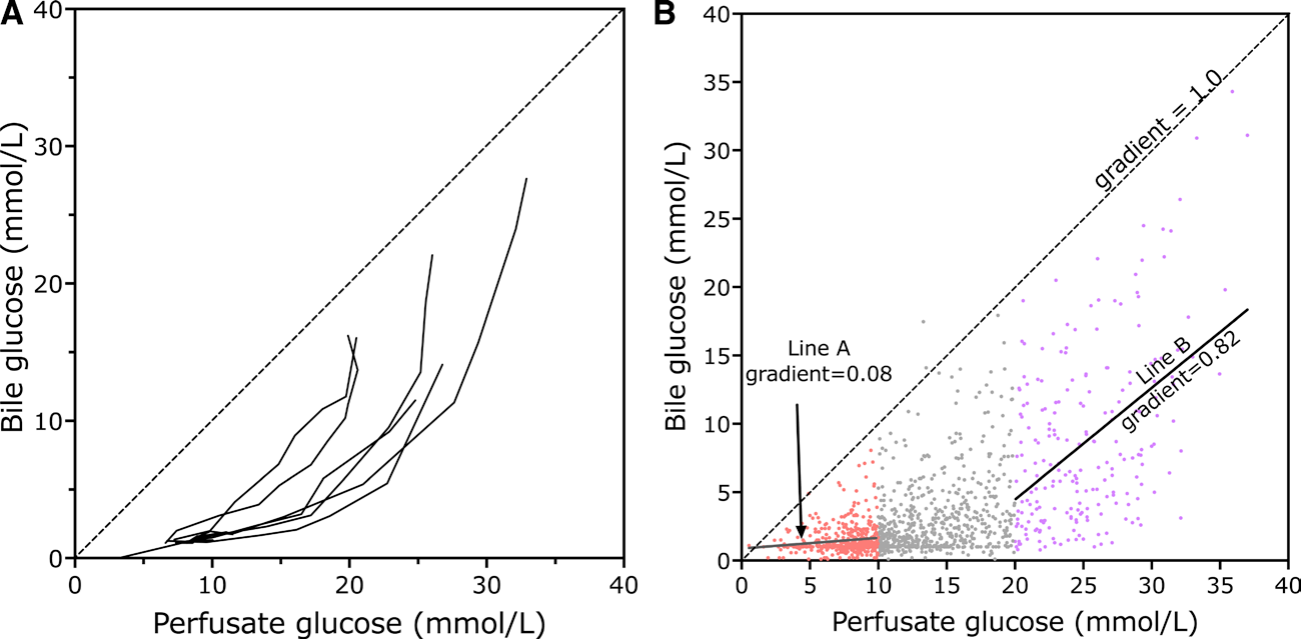
Bile and perfusate glucose concentrations for transplanted livers. A, The bile and perfusate glucose concentrations at serial time points for an illustrative number of transplanted livers that did not develop cholangiopathy joined by a single line. These curves represent the change over time as perfusate concentration falls. B, A scatter plot of individual perfusate/bile points of all the 200 transplanted livers, with 2 regression lines drawn. Line B is the regression line for perfusate glucose values >20 mmol/L; the slope of 0.82 implies that very little is reabsorbed when the perfusate glucose is >20 mmol/L. Line A is the regression line for perfusate values <10 mmol/L; the gradient of 0.08 implies that most of the glucose is reabsorbed from bile when the perfusate glucose is <10 mmol/L. The dotted line (gradient = 1.0) represents the line of identity along which the points would be expected to lie if bile and perfusate glucose concentrations were the same (after Guzelian and Boyer, 1974).^13^

Figure 4A shows 6 example livers to illustrate that as the perfusate glucose concentration rises beyond a varying threshold of between 5 and 20 mmol/L, glucose starts to appear in the bile at increasing concentrations. Considering all the transplanted livers, it can be seen from Figure 4B that at low perfusate concentrations (<10 mmol/L) the regression line of perfusate glucose concentrations is 0.078, suggesting nearly all the glucose is being pumped out of bile. When the perfusate concentration is >20 mmol/L the regression line is virtually parallel to the line of equivalence (ratio 0.82), such that with increasing perfusate glucose concentrations there is the same increase in bile glucose. This suggests that the process for resorbing glucose from bile has become saturated, and excess glucose remains in bile, in a similar manner to that described by Guzelian and Boyer in an animal model.^13^

### Calcium and Chloride

The concentration of chloride in bile was greater than perfusate, with occasional livers where it was substantially higher. The most outlying liver had a bile chloride peak at 202 mmol/L while the perfusate chloride was 101 mmol/L; this liver developed cholangiopathy requiring retransplantation. Nineteen other livers with chloride ratios >1.25 had no adverse biliary sequelae. The concentration of calcium in the bile was lower than perfusate for most livers; where this was not the case, there was no obvious clinical correlation. The absolute concentration of both chloride and calcium for most livers was very closely related to the perfusate concentrations.

### Chemistry of Livers Developing Cholangiopathy

**FIGURE 5.**
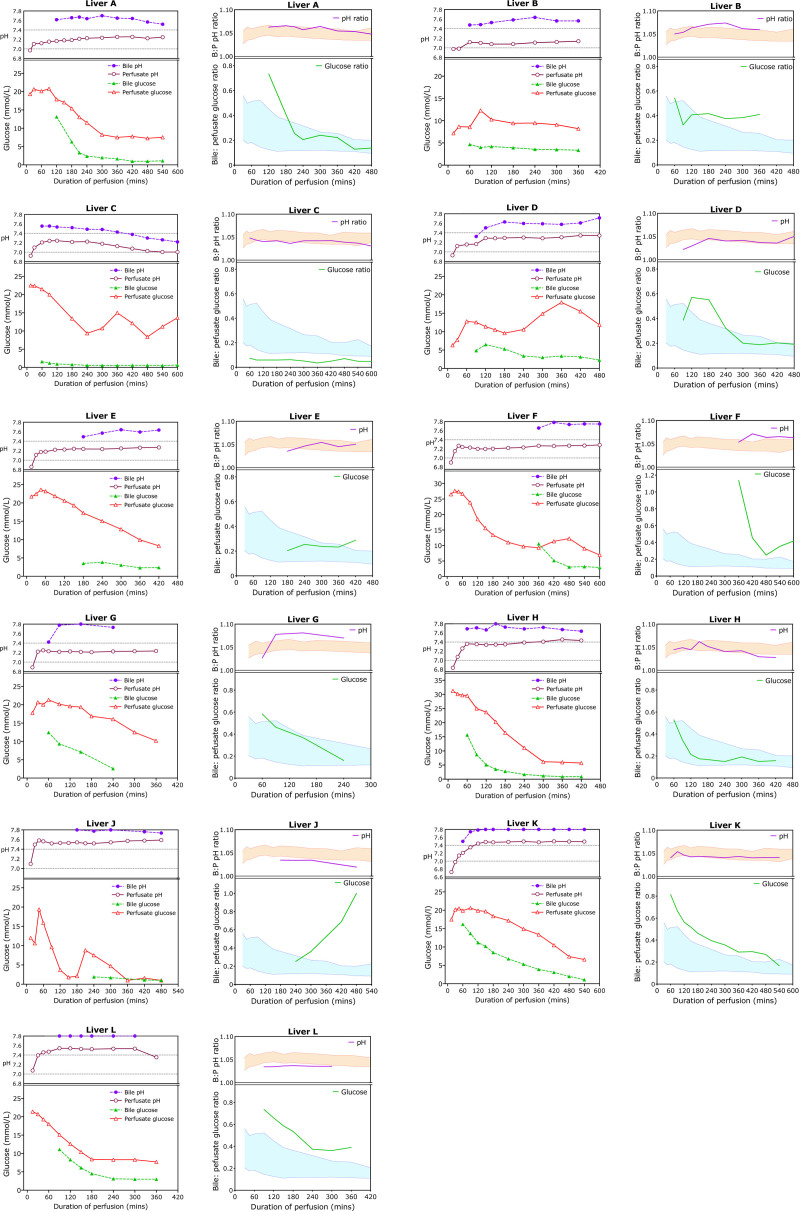
Perfusate and bile glucose and pH trends and ratios during perfusion of livers that went on to develop cholangiopathy of peripheral bile ducts. Eleven pairs of graphs showing the bile and perfusate pH and glucose in the left panel of each pair, and the ratio of bile:perfusate pH and glucose concentrations in the right panel of each pair. The graph of ratios has 2 shaded areas, the upper orange one showing the interquartile range of all pH ratios, whereas the lower shaded area represents the interquartile range of all glucose ratios. As can be seen from the pairs of graphs, these 11 livers fall within the common range of values or exceed them in terms of acceptability. Note that the curves for bile glucose and pH do not start at the outset of perfusion but at various time points as perfusion continues depending on when bile production began.

Figure 5 illustrates the bile and perfusate pH and glucose concentrations and ratios for the 11 livers developing NAS. Liver B was unique in having the highest biliary chloride of any of the 300 perfused livers in which it was measured including those perfused for research; it could not be measured in 14 cases because of lack of reagent. It achieved a bile pH >7.6 and maximum bile:perfusate glucose difference of 8.3. Liver C had the lowest bile glucose and a maximum bile:perfusate difference of 19.9 mmol/L but never achieved a bile pH >7.55, although the perfusate pH was lower than normal. The remaining livers all met ≥1 of the bile parameters we were using at the time, namely a bile pH >7.5 and bile glucose ≤3 mmol/L or >10 mmol/L less than perfusate glucose.^7^

### Profiles of Nontransplanted Livers

**FIGURE 6.**
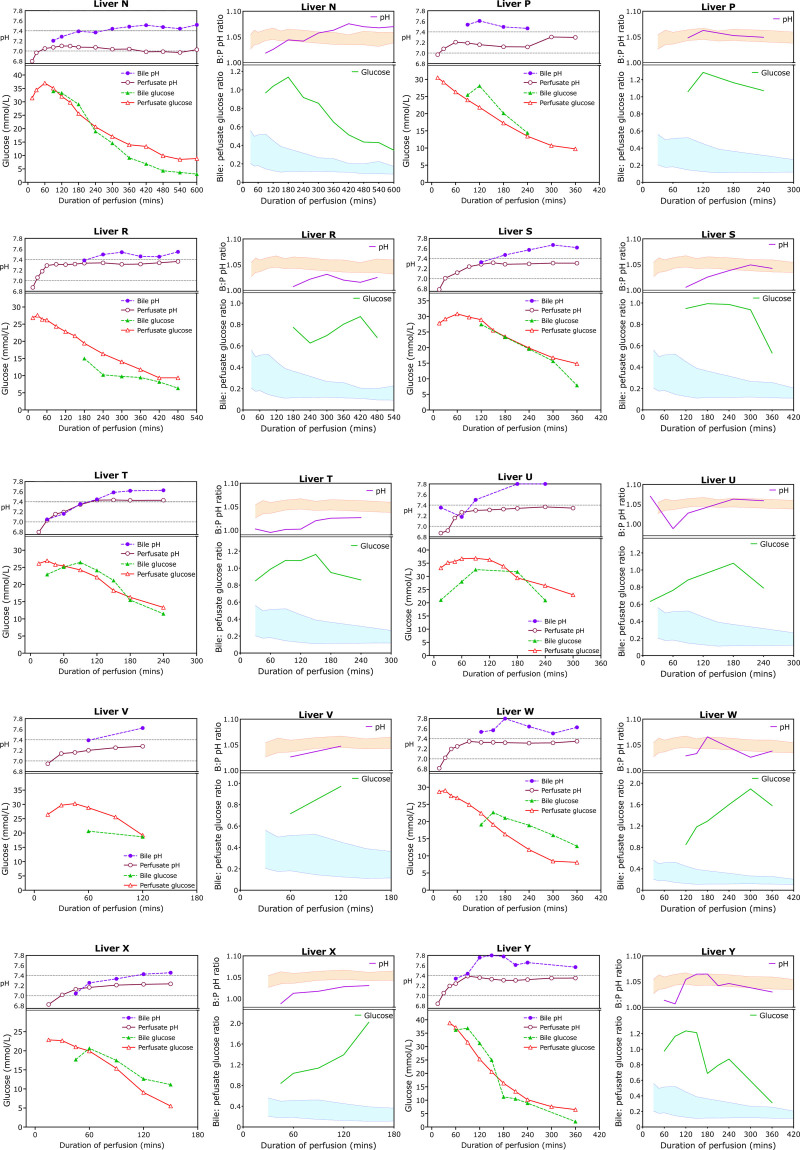
Perfusate and bile glucose and pH trends during perfusion of livers that were declined on the basis of bile chemistry. Similar to Figure 5, this figure shows concentrations on the left and ratios on the right for 10 pairs of livers selected as the first 10 in the series to be declined on biliary criteria. As can be seen, the bile pH and glucose are very abnormal and mimic perfusate values suggesting failure of cholangiocyte secretion and absorption.

Eighty-one livers were not transplanted because they were deemed to have poor hepatocellular function (ALT >6000 U/L; lactate not falling, glucose not falling, ongoing requirement for bicarbonate supplementation to maintain a pH of >7.2) or poor cholangiocyte function (bile pH <7.5, glucose not significantly lower than perfusate) or otherwise were poorly perfused; in addition 2 were not transplanted locally because the recipients died before implantation. The bile and perfusate concentrations and bile:perfusate concentration ratios for the first 10 livers declined wholly or in part based on bile glucose and pH are shown in Figure 6. The lack of difference in bile and perfusate glucose is apparent in most cases, with the lag in a fall in bile glucose concentration visible in many of the livers (livers N, P, T, U, W, X, Y) reflecting the slow bile production and dead space in the bile ducts and bile drainage tube.

## DISCUSSION

NESLiP is becoming increasingly common, and in this article, we describe the electrolyte chemistry of bile and changes in bile and perfusate analyte concentrations during NESLiP to aid clinicians in interpreting changes in their own practice. We have shown that the concentrations of sodium, potassium, and chloride in bile are slightly higher than perfusate, whereas that of calcium is lower. Glucose was much lower, as was the hydrogen ion concentration, with pH and bicarbonate higher than perfusate.

The glucose concentrations are notable in that they confirm, we believe for the first time in human livers, that glucose reabsorption from bile saturates, something demonstrated in rats 50 y ago by Guzelian and Boyer.^13^ Glucose resorption from the bile is thought to be primarily by a sodium-dependent glucose cotransporter 1, and this works in conjunction with the glucose transporter 1 on the basolateral plasma membrane of the cholangiocyte, which returns the glucose to the circulation.^2,14^ Absorption of glucose from bile will also form an osmotic gradient favoring the reabsorption of water from bile, so it is possible that in the presence of high perfusate glucose, with spill over into the bile, the bile will contain more water, and thus its volume is increased.

The saturable nature of glucose absorption mirrors glucose reabsorption by renal tubules, where high blood glucose results in glycosuria. Our data suggest that the threshold concentration that exceeds the absorptive capacity of cholangiocytes varies from liver to liver, much as the threshold for tubular loss in the kidneys varies from person to person. Figure 2 suggests that DCD livers appear less able to reabsorb glucose, possibly related to more severe cholangiocyte dysfunction because of greater ischemic injury. The degree to which the impaired resorption is reversible is unclear, and likewise, it is unclear whether it represents irreversible structural injury of the bile duct, which will later manifest as cholangiopathy. This questions the reliability of impaired glucose reabsorption as a measure of bile duct viability.

We and others have suggested that bile glucose may be a valuable discriminator between viable and nonviable cholangiocytes, that is, those cells lining the bile ducts destined to develop strictures.^7,8,15^ Based on our early experience, we had proposed that a bile glucose concentration <3 mmol/L, or 1 that was >10 mmol/L lower than perfusate glucose, suggested viable cholangiocytes.^7^ Matton et al observed histological damage of common hepatic ducts and common bile ducts and showed that bile duct injury was less common in livers with a bile:perfusate glucose ratio <0.67 and bile glucose <16 mmol/L. Figure 5 shows that all the livers in our series that developed cholangiopathy had a ratio <0.67, and usually much lower; they also all had bile glucose <16 mmol/L. Only liver B of the 10 that developed cholangiopathy failed our own bile glucose criteria, with lowest glucose of 3.4 mmol/L and bile:perfusate glucose difference of <10 mmol/L.

The other commonly quoted criterion relates to the ability of cholangiocytes to produce an alkali bile. This involves slightly different mechanisms in different species, the end result being an accumulation of bicarbonate in bile.^2^ In man, the secretion of bicarbonate is believed to involve chloride secretion into bile by the cystic fibrosis transmembrane conductance regulator (CFTR), and this chloride ion is then absorbed back into the cholangiocyte by the anion exchange pump 2 (AE2) in exchange for bicarbonate being pumped into bile. CFTR is the same gene that is mutated in cystic fibrosis and is under the control of secretin. A second pathway for chloride secretion via calcium-activated channels has been proposed, suggesting that the CFTR pathway might play a more regulatory role than being a primary channel, possibly through the release of ATP into the bile.^2,16^ The resulting high concentration of bicarbonate is thought to be protective to the bile duct, by preventing desialylation of cholangiocyte glycocalyx by bile acids, the so-called “bicarbonate umbrella.”^17^

Figure 2 shows that the bile produced during NESLiP is more alkali than perfusate, with a higher pH and higher bicarbonate content (see also Figure 3). Matton et al proposed a bile pH of >7.48 and a bicarbonate of >18 mmol/L to indicate a viable biliary tree, whereas we had suggested a pH of >7.5.^7,8^ All 11 livers that developed cholangiopathy in our series had a peak bile pH of >7.5, and in all but 2 livers, it was >7.6 (Figure 5; **Table S1, SDC,** http://links.lww.com/TP/C973). Only 4 of the 11 had bile bicarbonate below the Matton threshold of 16 mmol/L. Of the 200 livers studied that did not develop cholangiopathy, 29 (14.5%) did not achieve bile bicarbonate >16.0 mmol/L.

Chloride chemistry is also of note. Although the median bile chloride was just higher than perfusate chloride, there were some livers with high concentrations of chloride in bile. The most abnormal was liver B, which had a peak bile chloride of 202 mmol/L, double that of the perfusate. This suggests impairment of the AE2 pump, which should resorb chloride from bile in exchange for bicarbonate, and this would account for the low bile bicarbonate seen in this liver. This pattern of abnormality suggests that the AE2 and CFTR pumps are on cholangiocytes that are spatially separate because if both pumps were on the same cholangiocyte, one would expect both to be affected equally by disruption of that cholangiocyte, with no net change in chloride. Similarly, the ability to produce alkali bile but inability to absorb glucose also suggests that cholangiocytes with these functions are spatially separated along the bile ducts and experience different ischemic insults.

The other biochemical variable of note is the lactate, which was higher than perfusate as bile production began but became the same as perfusate in most cases. In 6 of the 11 cases, the bile lactate was at some point either the same as or less than that of the perfusate. It did not appear to be an indicator of impaired bile duct viability.

What we have detailed in this article is the range of bile chemistry during NESLiP as measurable on a near-patient blood gas chemical analyzer; whether these are “normal” values in ischemically compromised livers is unclear. We cannot quote a normal range of bile analytes because of the dependence of bile concentration on perfusate concentration. We have also highlighted the chemical changes in cases that developed cholangiopathy affecting peripheral ducts. These included cases that conformed to the published viability criteria, suggesting that the criteria have a significant false-negative rate, that is, that apparently normal bile chemistry, as defined in the criteria, can be falsely reassuring. This is probably explained by the fact that, when it manifests, ischemic cholangiopathy is often distributed randomly throughout the liver to a greater or lesser degree, as opposed to the entire biliary tree being affected. This would conform with the putative ischemic nature of the bile duct injury, as suggested by us and others, as being a consequence of multiple microthrombi-associated infarcts scattered throughout the ducts.^18-20^ It is likely that the remaining liver has sufficient functioning cholangiocytes to produce bile with a chemical composition that meets published criteria for transplantation. Tightening up the criteria will result in good livers being unused because it is possible that some of the observed early dysfunction of cholangiocytes is related to ischemia and reperfusion rather than irreversible microthrombi-associated infarction. Ignoring biliary chemistry altogether, as was done in the VITTAL study, runs the risks of severe biliary complications necessitating retransplantation.^21^ In addition, it should be acknowledged that cholangiocyte chemistry will not detect strictures in the first- and second-order ducts, because the cholangiocytes here are more concerned with the production of mucus and not electrolyte manipulation of bile. A viscometric assessment of mucus content would be more appropriate to detect abnormalities here, although these ducts are accessible by cholangioscopy and can be assessed that way.

We now believe that it is more important to focus on treating the presumed etiological cause of NAS, namely ischemia-induced microthrombi,^22,23^ than to try to predict which livers are likely to develop cholangiopathy based on bile chemistry. Our recent work suggests that D-dimer release during NESLiP (and probably during hypothermic oxygenated perfusion) reflects an underlying burden of microthrombi and can predict adverse transplant outcomes, including cholangiopathy.^23^ The fibrin responsible for D-dimer release appears to accumulate during cold storage but may also be present in part at the time of retrieval.

In summary, the chemistry of bile can provide useful insight into cholangiocyte function. This study has provided evidence for spatially distinct cholangiocyte functions along the bile duct and confirmed the observations in rodents that glucose reabsorption is via a saturable pump. Current published biliary viability criteria based on bile chemistry fail to detect all incidences of cholangiopathy but may still be valuable in identifying livers with very abnormal cholangiocyte function where the risk of cholangiopathy is high. Rather than trying to further define criteria to predict cholangiopathy, we believe attention should be turned to targeting the microthrombi, which appear to be the cause of cholangiopathy. Although microthrombi may be cleared using thrombolysis during NESLiP,^20,23^ it would be most desirable to research the trigger for microthrombi formation and prevent their formation during cold storage.

## ACKNOWLEDGMENTS

The authors acknowledge the help of their surgical and hepatology colleagues in conducting these studies, and they are grateful to Gareth Hayman, who facilitated the perfusions. They are indebted to the donors who donated their livers for transplantation into others and who were the subject of this study.

The Human Research Tissue Bank is supported by the NIHR Cambridge Biomedical Research Centre.

## Supplementary Material


