# Supplementary material for: Bile Chemistry During Ex Situ Normothermic Liver Perfusion Does Not Always Predict Cholangiopathy
Source: Transplantation. 2024 Feb 27;108(6):1383–93. doi: 10.1097/TP.0000000000004944 (PMC11115455; doi:10.1097/TP.0000000000004944)
Supplement: Supplementary file 1 [file tpa-108-1383-s001.pdf]

Bile chemistry during *ex situ* normothermic liver perfusion does not always predict cholangiopathy: Supplementary data

Authors

Christopher J. E. Watson MA MD BChir [1, 2, 3, 4]

Rohit Gaurav MB BS MS[4]

Lisa Swift [4]

Corrina Fear [4]

Michael E.D. Allison MB BS PhD [4,8]

Sara S. Upponi MB BS

Rebecca Brais BA MB[7]

Andrew J Butler MA MB MChir

Supplementary Table 1. Characteristics of livers developing cholangiopathy in our series.

Liver	DCD / DBD	Donor age	Withdrawal period	FWIT	Asystolic period	Cold ischaemic time	US DRI	UK DLI	Highest bile pH	Highest bicarbonate	Lowest glucose	Lowest B:P glucose ratio	Largest B:P glucose difference
A	DCD	53	16	N/R	16	432	2.51	2.05	7.70	16.4	<1.0	0.15	9.7
B	DCD	69	17	N/R	11	343	3.28	2.77	7.64	11.0	3.4	0.32	8.3
C	DBD	42	0	0	0	478	1.47	1.20	7.55	15.3	<1.0	0.04	19.9
D	DCD	62	11	18	15	404	2.85	2.02	7.71	16.1	2.3	0.19	14.6
F	DCD	69	15	14	12	396	3.20	2.21	7.78	13.5	2.3	0.20	10.6
E	DCD	55	14	9	8	544	2.75	2.49	7.64	14.6	2.3	0.20	13.7
G	DBD	53	0	0	0	520	2.07	1.42	7.90	23.7	2.6	0.16	13.5
H	DCD	19	15	20	10	441	1.74	1.15	7.86	20.1	0.9	0.15	18.6
K	DCD	37	11	N/R	11	496	1.84	1.39	>7.8	>19.5*	1.1	0.17	10.4
J	DCD	58	111	N/R	9	404	2.78	1.78	7.59	31.8	1.0	0.25	5.6
L	DCD	32	13	N/R	13	371	2.23	1.49	>7.8	*	1.9	0.28	5.3

DBD: Donation after brain death; DCD: Donation after circulatory death; FWIT: Functional warm ischaemic time; N/R: Not recorded; US DRI: United States Donor Risk Index; UK DLI: United Kingdom Donor liver index; B:P Bile:perfusate; * pH out of range so bicarbonate could not be calculated, but would be high. All concentrations are in mmol/L. All times are in minutes.

Definitions: Cholangiopathy: Peripheral bile duct strictures not associated with hepatic artery thrombosis; Withdrawal period: withdrawal of treatment to asystole; Functional warm ischaemic time: systolic blood pressure <50mmHg to cold in situ perfusion;. Asystolic period: circulatory arrest to cold in situ perfusion.

Supplementary table 2. Characteristics of the 72 transplanted livers that did not develop cholangiopathy

	**Cholangiopathy n=11**		**No cholangiopathy n=200**	
DCD / DBD	9 DCD, 2 DBD		110 DCD, 90 DBD	
Donor age (years)	53	(19, 37, 62, 69)	48	(11, 31, 57, 78)
Withdrawal period (mins)†	14	(0, 11, 16, 111)	14	(0, 11 ,17, 104)
Asystolic period (mins)†	11	(5, 8, 13, 16)	12	(6, 11, 14, 25)
Cold ischaemic time (mins)	432	(343, 396, 496, 544)	408	(91, 335, 492, 910)
US DRI	2.5	(1.5, 1.8, 2.9, 3.3)	2.0	(1.1, 1.6, 2.4, 3.6)
UK DLI	1.8	(1.2, 1.4, 2.2, 2.8)	1.5	(0.6, 1.0, 2.0, 2.8)
UK DCD risk	5 low; 3 high; 1 futile		63 low; 38 high; 9 futile	
2 hour lactate (mmol/L)	0.3	(0.1, 0.1, 0.9, 3.2)	1.0	(0.1, 0.4, 1.8, 5.1)
2 hour ALT (IU/L)	1800	(507, 1291, 4161, 4816)	1347	(111, 666, 2179, 10439)
Bile volume at 4h (mls)	16.1	(0, 6.5, 29.9, 55.4)	21.0	(0, 9.0, 34.7, 110.5)
Highest bile pH	7.71	(7.55, 7.64, >7.8, >7.9)	7.80	(7.30, 7.74, 7.80, 8.00)
Lowest bile glucose(mmol/L)	1.9	(0.9, 1.0, 2.3, 3.4)	1.0	(0.1, 1.0, 1.7, 17.8)
Largest Bile:Plasma glucose difference (mmol/L)	10.6	(5.3, 8.3, 14.6, 19.9)	12.5	(0, 9.0, 17.1, 29.2)
MEAF	5.1	(1.0, 2.6, 5.5, 7.9)	3.7	(0.3, 2.5, 5.7, 9.4)

Data are median (minimum, lower interquartile range, upper interquartile range, maximum).

† Withdrawal of treatment to circulatory arrest in DCDs only. Excludes livers that underwent *in situ* normothermic regional perfusion before NESLiP.

MEAF: Model for early allograft function score [1]; US DRI: United States Donor Risk Index [2,3];
UK DLI: United Kingdom Donor Liver index [4]. UK DCD Risk: UK DCD Risk Score [5]

References

1. Pareja E, Cortes M, Hervas D, et al. A score model for the continuous grading of early allograft dysfunction severity. *Liver Transpl*. Jan 2015;21(1):38-46. doi:10.1002/lt.23990

2. Feng S, Goodrich NP, Bragg-Gresham JL, et al. Characteristics Associated with Liver Graft Failure: The Concept of a Donor Risk Index. *Am J Transplant*. 2006;6:783-90.

3. Schaubel DE, Sima CS, Goodrich NP, Feng S, Merion RM. The survival benefit of deceased donor liver transplantation as a function of candidate disease severity and donor quality. *Am J Transplant*. Feb 2008;8(2):419-25. doi:10.1111/j.1600-6143.2007.02086.x

4. Collett D, Friend PJ, Watson CJ. Factors Associated With Short- and Long-term Liver Graft Survival in the United Kingdom: Development of a UK Donor Liver Index. *Transplantation*. Apr 2017;101(4):786-792. doi:10.1097/TP.0000000000001576

5. Schlegel A, Kalisvaart M, Scalera I, et al. The UK DCD Risk Score: A new proposal to define futility in donation-after-circulatory-death liver transplantation. *J Hepatol*. Mar 2018;68(3):456-464. doi:10.1016/j.jhep.2017.10.034
